# Assessment of the In Vitro Antischistosomal Activities of the Extracts and Compounds from Solidago Microglossa DC (Asteraceae) and Aristolochia Cymbifera Mart. & Zucc. (Aristolochiaceae) 

**DOI:** 10.1155/2020/1726365

**Published:** 2020-09-29

**Authors:** Poliana da Silva Costa, Ohana Oliveira Zuza da Silva, Danilo de Souza Costa, Lara Aparecida de Oliveira Silva, Priscila de Faria Pinto, Marcos Paulo Nascimento da Silva, Josué de Moraes, Ademar A. Da Silva Filho

**Affiliations:** ^1^Faculty of Pharmacy, Department of Pharmaceutical Sciences, Federal University of Juiz de Fora, Juiz de Fora, MG 36036-900, Brazil; ^2^Department of Parasitology, Microbiology and Immunology, Biological Sciences Institute, Federal University of Juiz de Fora, Juiz de Fora, MG 36036-900, Brazil; ^3^Núcleo de Pesquisa em Doenças Negligenciadas, Universidade Guarulhos, Guarulhos, SP 07025-000, Brazil

## Abstract

Schistosomiasis, caused by helminth flatworms of the genus *Schistosoma*, is a neglected tropical disease that afflicts over 230 million people worldwide. Currently, treatment is achieved with only one drug, praziquantel (PZQ). In this regard, the roots of *Solidago microglossa* (Asteraceae) and *Aristolochia cymbifera* (Aristolochiaceae) are popularly used as anthelmintic. Despite their medicinal use against helminthiasis, such as schistosomiasis, *A*. *cymbifera*, and *S*. *microglossa* have not been evaluated against *S*. *mansoni*. Then, in this work, the in vitro antischistosomal activity of the crude extracts of *A*. *cymbifera* (Ac) and *S*. *microglossa* (Sm) and their isolated compounds were investigated against *S*. *mansoni* adult worms. Sm (200 *μ*g/mL) and Ac (100–200 *μ*g/mL) were lethal to all male and female worms at the 24 h incubation. In addition, Sm (10–50 *μ*g/mL) and Ac (10 *μ*g/mL) caused significant reduction in the parasite's movements, showing no significant cytotoxicity to Vero cells at the same range of schistosomicidal concentrations. Confocal laser scanning microscopy revealed that Sm and Ac caused tegumental damages and reduced the numbers of tubercles of male schistosomes. Chromatographic fractionation of Sm leads to isolation of bauerenol, *α*-amirin, and spinasterol, while populifolic acid, cubebin, 2-oxopopulifolic acid methyl ester, and 2-oxopopulifolic acid were isolated from Ac. At concentrations of 25–100 *μ*M, bauerenol, *α*-amirin, spinasterol, populifolic acid, and cubebin showed significant impact on motor activity of *S*. *mansoni*. 2-oxopopulifolic acid methyl ester and 2-oxopopulifolic acid caused 100% mortality and decreased the motor activity of adult schistosomes at 100 *μ*M. This study has reported, for the first time, the in vitro antischistosomal effects of *S*. *microglossa* and *A*. *cymbifera* extracts, also showing promising compounds against adult schistosomes.

## 1. Introduction

Schistosomiasis, a chronic parasitic disease caused by helminth flatworms of the genus *Schistosoma*, afflicts over 250 million people worldwide [1–3], being the second-most important human parasitic disease in terms of public health [1, 4]. There is currently no vaccine for schistosomiasis, and chemotherapy relies on one drug only, praziquantel (PZQ) [2, 5]. Although PZQ is safe, it exhibits lack of activity against juvenile worms and limited effects on liver and spleen lesions, and its use over the last decades may contribute to emerging PZQ-resistance development [3, 6]. Therefore, the lack of any other effective and safe schistosomicidal compounds has raised the urgent need for new antischistosomal drugs that could either complement or replace PZQ chemotherapy [7]. As a result, the search for antischistosomal compounds, especially from natural sources, has been increased [2].

In this regard, several *Solidago* (Asteraceae) species are used in folk medicines of all over the world for many medicinal purposes, including as antiparasitic and antiseptic [8, 9]. *Solidago microglossa* De Candolle (Asteraceae), synonymy *S*. *chilensis* Meyen, is a medicinal plant known as “arnica-do-campo” and “erva-lanceta” that possess a very widely popular therapeutic applications in South America, including as anti-inflammatory [10, 11] and anthelmintic [12, 13]. Due to its high medicinal importance in Brazil, since 2009, *S*. *microglossa* is included in the National List of Medicinal Plants of Interest to the Brazilian Unified Health System (RENISUS) [10, 11, 13].

Also, in Brazil, mainly in the South States, the roots decoctions of *S*. *microglossa* are popularly used as anthelmintic for the complementary and alternative treatment of some parasitic diseases [13–16]. Although *S*. *microglossa* roots are employed for the treatment of helminthiasis, this popular indication against helminths, such as *Schistosoma*, which arise from the traditional knowledge, has not been supported by any scientific evidence so far.

In addition, studies have shown that the roots decoctions of some *Aristolochia* species known as “cipó-mil-homens,” such as *A*. *triangularis* and *A*. *cymbifera*, with suggested oral doses varying of 0.1 to 2 mL up to three times daily, have been used in the traditional medicine of South America as anthelmintic and for the treatment of malaria and general infectious [8, 9, 14–19]. *A*. *cymbifera* Mart. & Zucc (Aristolochiaceae), synonymy *A*. *esperanzae* Kuntze, is a medicinal plant used in Brazilian folk medicine for the treatment of infectious diseases, malaria, wounds, fever, diarrhea, snakebite, and as anthelmintic [8, 9, 14, 16–19]. Previous studies showed antibacterial, antifungal, trypanocidal, and antileishmanial activities for *A*. *cymbifera* extracts [20–22]. Due to its significance in the traditional medicine, *A*. *cymbifera* is included in the first edition of the Brazilian Official Pharmacopoeia [23]. Nowadays, the roots of *A*. *cymbifera* are sold in several Brazilian markets, either alone or in combination with other plants, as herbal remedies for the treatment of helminthiasis, such as schistosomiasis and general infections [8, 9, 17, 19]. However, despite its medicinal use against helminths, *A*. *cymbifera* have not been evaluated against *Schistosoma*.

Then, based on their traditional use as anthelmintic, we wondered whether the roots of *S*. *microglossa* and *A*. *cymbifera* and their isolated compounds possess effects against *S*. *mansoni*. Thus, in this work, we evaluated the in vitro antischistosomal effects of the crude extracts and isolated compounds from the roots of *S*. *microglossa* and *A*. *cymbifera*.

## 2. Materials and Methods

### 2.1. Plant Material and Extraction

This study was developed in line with Brazilian Federal Law number 13.123/2015 on Access to Genetic Heritage, registered under number AE32DB3. Roots of *S*. *microglossa* (650 g) and *A*. *cymbifera* (1300 g) were collected at Faculty of Pharmacy's garden from the Federal University of Juiz de Fora. Exsiccates of the plant species were deposited in the Herbarium Leopoldo Krieger of the Federal University of Juiz de Fora, MG, Brazil, under the numbers #64488 (*S*. *microglossa*) and #50054 (*A*. *cymbifera*). After collected, roots were dried at 40°C, pulverized, and extracted, by maceration, using ethanol : water (8 : 2 v/v) as solvent. Next, the solvent was removed under reduced pressure to yield 15 g of the crude hydroalcoholic extract of the roots of *S*. *microglossa* (Sm) and 40 g of the hydroalcoholic extract from the roots of *A*. *cymbifera* (Ac).

### 2.2. Isolation and Identification of Compounds

The crude extract Sm (10 g) was chromatographed over silica gel (70–230 mesh, Merck) under the vacuum liquid chromatography system (VLC, glass columns with 5–10 cm id), using hexane: EtOAc mixtures in increasing proportions as eluent, furnishing nine fractions. The resulting fraction IV (hexane: EtOAc 85 : 15; 1.2 g) was submitted to classic column chromatography over silica gel, using hexane: EtOAc in increasing proportions as eluent, furnishing the compounds **1** (0.23 g), **2** (0.20 g), and **3** (0.05 g).

In addition, the crude extract Ac (35 g) was firstly chromatographed over silica gel (VLC system, 70–230 mesh, Merck glass columns with 5–10 cm i.d) using hexane: EtOAc mixtures to furnish 8 fractions. Next, fraction II (Ac. II, hexane: EtOAc 9 : 1; 10.2 g) was additionally chromatographed over VLC system with silica gel with hexane: EtOAc mixtures as eluent, giving 6 subfractions, affording **4** (1.7 g, from fraction Ac. II.d). Also, fraction V was submitted to flash chromatography using DCM: EtOAc (97 : 3, v/v) as eluent, furnishing the compound **5** (0.07 g). Fraction VI was submitted to semipreparative reverse-phase HPLC purification (HPLC-DAD Waters 2998), binary HPLC pump, column ODS 250 × 10 mm, 5 *μ*m, UV-DAD detector at 220 nm) using MeOH-H_2_O 75 : 25 v/v as mobile phase, affording **6** (0.02 g) and **7** (0.01 g).

Chemical structures of all compounds were established by ^1^H- and ^13^C-NMR analysis in comparison with the literature. ^1^H- and ^13^C- NMR spectra were recorded in CDCl_3_ solutions on a Bruker 500 Advance spectrometer (500 MHz for ^1^H NMR and 125 MHz for ^13^C NMR) with chemical shifts (*δ*) reported in parts per million (ppm) relative to trimethylsilane (TMS) as internal standard and coupling constants (*J*) in Hertz (Hz). The purity of all isolated compounds was predicted to be higher than 95% by ^13^C- and ^1^H-NMR data analysis.

Compound **1** (baurenol): the NMR spectroscopic data are according to the literature [24]. ^1^H NMR (500 MHz, CDCl_3_) *δ* (ppm): 3.24 (1H, s, OH); 5.40 (2H, m, H-7 and H8). ^13^C NMR (125 MHz, CDCl_3_) *δ* (ppm): 37.0 (C-1); 27.8 (C-2); 79.4 (C-3); 39.0 (C-4); 50.5 (C-5); 24.3 (C-6); 116.5 (C-7); 145.4 (C-8); 48.3 (C-9); 35.3 (C-10); 16.9 (C-11); 32.5 (C-12); 37.8 (C-13); 41.3 (C-14); 28.9 (C-15); 37.8 (C-16); 32.1 (C-17); 55.0 (C-18); 35.4 (C-19); 32.1 (C-20); 29.3 (C-21); 31.6 (C-22); 27.6 (C-23); 14.8 (C-24); 13.1 (C-25); 23.5 (C-26); 22.8 (C-27); 39.7 (C-28); 25.7 (C-29); 22.7 (C-30).

Compound **2** (*α*-amirin): the NMR spectroscopic data are according to the literature [25]. ^1^H NMR (500 MHz, CDCl_3_) *δ* (ppm): 4.5 (1H, s, OH); 5.12 (1H, dt, H-12). ^13^C NMR (125 MHz, CDCl_3_) *δ* (ppm): 38.1 (C-1); 27.8 (C-2); 79.4 (C-3); 39.0 (C-4); 55.0 (C-5); 18.5 (C-6); 32.5 (C-7); 41.3 (C-8); 48.3 (C-9); 37.0 (C-10); 23.8 (C-11); 124.5 (C-12); 139.7 (C-13); 41.6 (C-14); 28.2 (C-15); 26.3 (C-16); 33.8 (C-17); 59.2 (C-18); 38.9 (C-19); 39.8 (C-20); 31.4 (C-21); 41.6 (C-22); 29.0 (C-23); 15.7 (C-24); 15.8 (C-25); 16.9 (C-26); 23.5 (C-27); 28.8 (C-28); 16.9 (C-29); 21.5 (C-30).

Compound **3** (spinasterol): the NMR spectroscopic data are according to the literature [26]. ^1^H NMR (500 MHz, CDCl_3_) *δ* (ppm): 3.60 (1H, s, OH); 5.02 (1H, dd, *J*_1_ = 8.0 Hz and *J*_2_ = 15.0 Hz, H-23); 5.15 (2H, m, H-7 and H-22). ^13^C NMR (125 MHz, CDCl_3_) *δ* (ppm): 37.2 (C-1); 31.6 (C-2); 71.2 (C-3); 38.1 (C-4); 40.4 (C-5); 29.7 (C-6); 117.6 (C-7); 139.7 (C-8); 49.5 (C-9); 34.3 (C-10); 21.5 (C-11); 39.6 (C1-2); 43.4 (C-13); 55.2 (C-14); 23.1 (C-15); 28.6 (C-16); 56.0 (C-17); 12.2 (C-18); 13.1 (C-19); 40.9 (C-20); 21.5 (C-21); 138.3 (C-22); 129.5 (C-23); 51.4 (C-24); 32.0 (C-25); 21.2 (C-26); 21.2 (C-27); 25.5 (C-28); 12.4 (C-29).

Compound **4** (populifolic acid): the NMR spectroscopic data are according to the literature [27]. ^1^H NMR (500 MHz, CDCl_3_) *δ* (ppm): 0.71 (3H, s, H-20); 0.79 (3H, d, *J* = 6.5 Hz, H-17); 0.98 (3H, d, *J* = 7.2 Hz, H-16); 1.00 (3H, s, H-18); 1.57 (3H, d, *J* = 1.7 Hz, H-19); 5.18 (1H, s, H-3). ^13^C NMR (125 MHz, CDCl_3_) *δ* (ppm): 18.2 (C-1); 26.8 (C-2); 120.3 (C-3); 144.4 (C-4); 38.1 (C-5); 36.8 (C-6); 27.5 (C-7); 36.2 (C-8); 38.5 (C-9); 46,4 (C-10); 35.4 (C-11); 29.4 (C-12); 30.8 (C-13); 41.4 (C-14); 179.2 (C-15, C=O); 19.9 (C-16); 15.9 (C-17); 19.8 (C-18); 17.9 (C-19); 18.4 (C-20).

Compound **5** (cubebin): the NMR spectroscopic data are according to the literature [28]. ^1^H NMR (500 MHz, CDCl_3_) *δ* (ppm): 2.01 (1H, m, H-8); 2.43 (2H, m, H-7 and H-8′); 2.69 (1H, m, H-7′); 4.11 (1H, t, *J* = 7.8 Hz, H-9′a); 3.59 (1H, t, *J* = 7.8 Hz, H-9′b); 5.24 (1H, s, H-9); 5.94 (4H, s, 2x OCH_2_O); 6.63 (6H, m, H-2, H-5, H-6 and H-2′, H-5′, H-6′). ^13^C NMR (125 MHz, CDCl_3_) *δ* (ppm): 133.2 (C-1); 108.9 (C-2); 147.6 (C-3); 145.7 (C-4); 108.2 (C-5); 121.4 (C-6); 38.4 (C-7); 53.0 (C-8); 103.3 (C-9); 134.1 (C-1′); 109.1 (C-2′); 147.5 (C-3′); 145.9 (C-4′); 108.0 (C-5′); 121.7 (C-6′); 39.2 (C-7′); 45.8 (C-8′); 72.1 (C-9′); 100.8 (2 x OCH_2_O).

Compound **6** (2-oxopopulifolic acid methyl ester): the NMR spectroscopic data are according to the literature [29–31]. ^1^H NMR (500 MHz, CDCl_3_) *δ* (ppm): 0.65 (6 H, m, H-17 and H-20); 0.96 (3 H, d, *J* = 6.0 Hz, H-16); 1.06 (3H, s, H-19); 1.80 (3H, s, H-18); 3.67 (3H, s, OCH_3_); 5.65 (1H, s, H-3). ^13^C NMR (125 MHz, CDCl_3_) *δ* (ppm): 35.4 (C-1); 188.9 (C-2, C=O); 137.2 (C-3); 172.2 (C-4); 38.1 (C-5); 35.2 (C-6); 26.3 (C-7); 37.5 (C-8); 38.8 (C-9); 50.9 (C-10); 35.1 (C-11); 32.2 (C-12); 30.9 (C-13); 38.1 (C-14); 178.6 (C-15, CO_2_H); 19.9 (C-16); 15.2 (C-17); 18.4 (C-18); 19.6 (C-19); 17.3 (C-20); 26.3 (OCH_3_).

Compound **7** (2-oxopopulifolic acid): the NMR spectroscopic data are according to the literature [29–31]. ^1^H NMR (500 MHz, CDCl_3_) *δ* (ppm): 0.80 (3H, s, H-20); 0.96 (3H, d, *J* = 6.0 Hz, H-16); 1.10 (6H, s, H-17 and H-19); 1.87 (3H, s, H-18); 5.70 (1H, s, H-3); 7.60 (1H, s, COOH). ^13^C NMR (125 MHz, CDCl_3_) *δ* (ppm): 34.2 (C-1); 189.0 (C-2, C=O); 137.3 (C-3); 172.2 (C-4); 41.5 (C-5); 37.3 (C-6); 26.2 (C-7); 37.2 (C-8); 37.9 (C-9); 50.0 (C-10); 34.2 (C-11); 35.2 (C-12); 31.0 (C-13); 41.5 (C-14); 172.2 (C-15, CO_2_H); 20.0 (C-16); 15.4 (C-17); 17.8 (C-18); 18.3 (C-19); 17.3 (C-20).

### 2.3. Antischistosomal Assays

#### 2.3.1. Maintenance of the *S*. *mansoni* Life Cycle

*Schistosoma mansoni* (BH strain) was maintained by passage through *Biomphalaria glabrata*, as the intermediate host and Swiss female mice (Anilab, São Paulo, Brazil) as definitive host as previously described [32]. Both mice and snails were kept under environmentally controlled conditions (temperature, 25°C; humidity, 50%), with unrestricted access to rodent food and water.

#### 2.3.2. In Vitro Antischistosomal Assay

Seven weeks after infection, *S*. *mansoni* were removed from the hepatic portal system and cultured in RPMI 1640 culture medium (supplemented with 5% inactivated fetal calf serum and 100 U/mL penicillin and 100 *μ*g/mL streptomycin (Vitrocell, Campinas, SP, Brazil) at 37°C in an atmosphere of 5% CO_2_ until usage. For the determination of activity against adult schistosomes, all samples were initially tested at the concentration of 100 *μ*M (compounds) or 100 *μ*g/mL (extracts), using DMSO stock solutions (10 mM) diluted in supplemented RPMI 1640 medium within 24 flat bottom well plates (Tissue Culture Plastics, TPP, St. Louis, MO) with a final volume of 2 mL per well [33]. Samples were tested in triplicate with two worms of both sexes placed into each well. Wells with the highest concentration of DMSO in medium (0.5%) served as controls. Praziquantel (2 *μ*M) served as positive control. Parasites were kept for 48 h (37°C, 5% CO_2_), and their viability was assessed via microscopic readout (Leica Microsystems, Wetzlar, Germany) [34]. Next, samples presenting antischistosomal activity were tested at lower concentrations, as described above, and each experiment was performed at least three times [35].

#### 2.3.3. Microscopy Studies

Adult schistosomes were monitored by light microscopy using a Leica Microsystems EZ4E (Wetzlar, Germany) [34, 36]. Also, tegumental alteration and quantification of the number of tubercles were performed in adult male schistosomes for Sm and Ac (10, 25, 50, and 100 *μ*m) using a confocal laser scanning microscope (Laser Scanning Microscope, LSM 510 META, Carl Zeiss, Göttingen, Vertrieb, Germany). For confocal studies, after the occurrence of death, helminths were fixed in a formalin-acetic acid-alcohol solution and analyzed under a confocal microscope with autofluorescence excited at 488 nm and emitted light at 505 nm [36].

#### 2.3.4. Assessment of the Schistosome Egg Output

Sexual fitness of adult worms exposed to nonlethal concentrations of samples and parasites were monitored in order to determine the schistosome egg output by counting the number of eggs for five days using an inverted microscope, as previously described [37]. After 48 h of drug exposure and to analyze reversible effect on egg output, the medium containing samples was removed and worms were carefully rinsed with RPMI to prevent separation of the pairs. Then, worms were incubated continuously in the medium without drug and monitored for five days [37].

#### 2.3.5. Ethics Statement

All experiments were conducted in conformity with the Brazilian law for Guidelines for Care and Use of Laboratory Animals (Law 11790/2008). The protocol for experimental design was approved by the Comissão de Ética no Uso de Animais (CEUA), Brazil (Protocols ≠ CEUA, 11.794/08). Animal studies are reported in compliance with the ARRIVE guidelines.

### 2.4. In Vitro Cytotoxicity Studies

Mammalian Vero cells (African green monkey kidney fibroblast) used in this study were obtained from the American Type Culture Collection (ATCC CCL-81; Manassas, VA) and provided by Instituto Butantan (São Paulo, Brazil). Cytotoxicity of the samples was determined using the MTT assay [35]. The values of cytotoxic concentration reducing 50% of viable cells (CC_50_) were obtained using GraFit Version 5 software.

### 2.5. Statistical Analysis

The statistical tests were performed with using Graph Pad Prism software 5.0 (Graphpad software Inc., La Jolla, CA, USA). Significant differences were determined by one-way analysis of variance (ANOVA) and applying Tukey's test for multiple comparisons with a level of significance set at *P* < 0.05.

## 3. Results and Discussion

Schistosomiasis is a neglected disease with a huge impact in public health. Also, there is only one available drug to treat schistosomiasis, and due to the urgent need to identify new drugs, several natural compounds have been recently investigated against *S*. *mansoni* [2, 38]. In this regard, WHO encouraged the study and development of new pharmaceutical products on medicinal plants, especially in underdeveloped countries, as a relevant approach for the experimental treatment of schistosomiasis [39, 40].

In this context, we have highlighted the in vitro antischistosomal activity of *S*. *microglossa* and *A*. *cymbifera* extracts and their isolated compounds against *S*. *mansoni*, which have not been reported in the literature.

According to the literature [37, 39, 40], in vitro assays are essential tools to the initial selection of a potential anthelmintic drug. Then, after preparation, the crude extracts Sm and Ac were assayed against *S*. *mansoni*. Effects on mortality rate and motor activity of parasites after incubation with Sm and Ac, at concentrations of 10–200 *μ*g/mL, are shown in Table 1. Sm (200 *μ*g/mL) and Ac (100–200 *μ*g/mL) were lethal to all male and female worms at the 24 h incubation, while Sm (100 *μ*g/mL) and Ac (25–50 *μ*g/mL) caused death in half of adult worms and significant reduction in motor activity together with tegumental alterations. In addition, concentrations of 10–50 *μ*g/mL of Sm and 10 *μ*g/mL of Ac were not lethal to schistosomes but caused significant reduction in the parasite's movements (Table 1). Furthermore, Sm and Ac showed no significant cytotoxicity to Vero cells at the same range of schistosomicidal concentrations, as shown in Table 1.

**Figure 1:**
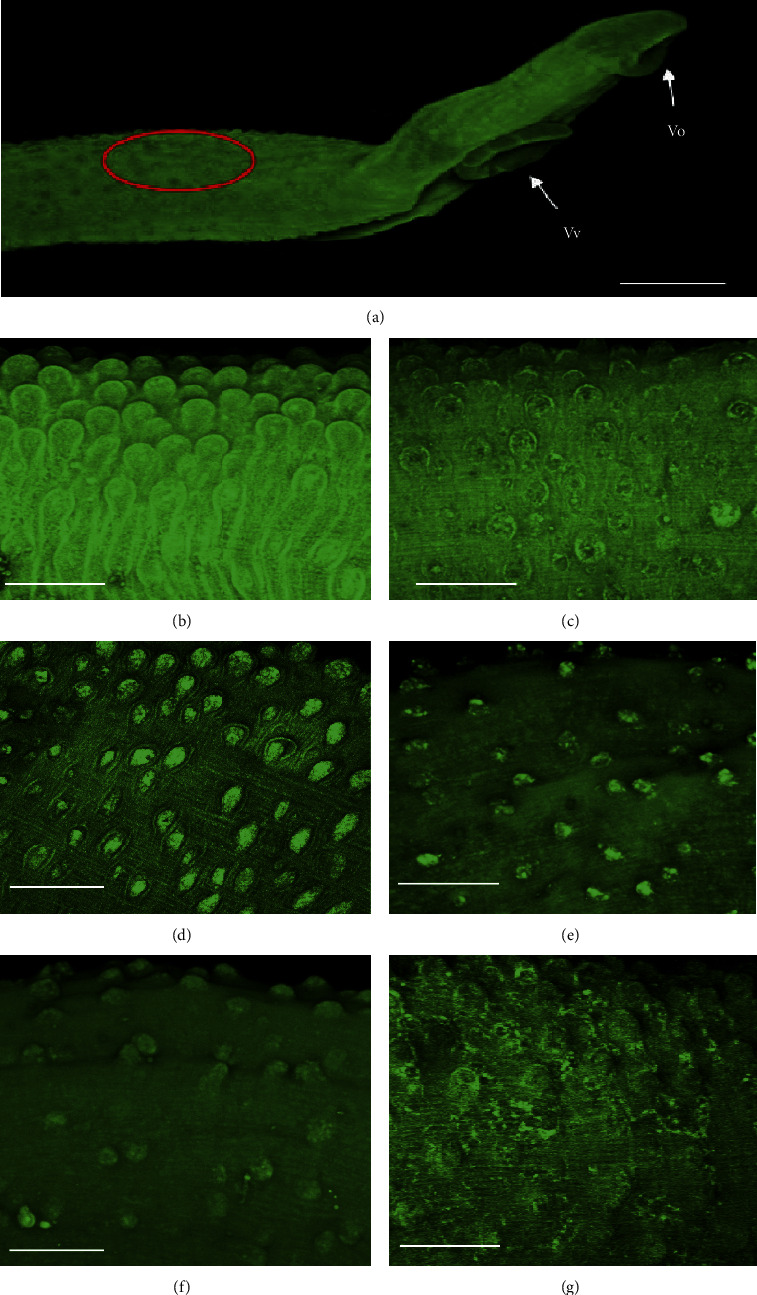
Confocal laser scanning microscopy observations of *S*. *mansoni* male worms after in vitro incubation with Sm and Ac. (a) General view of the anterior worm region showing, in red, the location where tegument was analyzed. (b) Control containing RPMI 1640 with DMSO 0.5%. (c) 2 *μ*M PZQ. (d) Sm 100 *μ*g/mL. (e) Sm 200 *μ*g/mL. (f) Ac 100 *μ*g/mL. (g) Ac 200 *μ*g/mL. Scale bars, 200 *μ*m.

In addition, given the importance of the worm's tegument in the action of new drugs, confocal laser microscopy studies were performed to evaluate morphological damages, tegument structures, and their alterations at the male surface of worms exposed to the plant extracts Sm and Ac (Figure 1). Along with dead, treatment with Sm (100–200 *μ*g/mL) and Ac (100–200 *μ*g/mL) (Figure 1) also caused evident damage at tegument of male schistosomes, in which destruction of tubercles was observed in a dose-dependent manner (Figure 1). Additionally, male adult schistosomes treated with Sm (200 *μ*g/mL) and Ac (200 *μ*g/mL) showed apparent rupture and/or disintegration of tubercles, which appeared eroded and deformed along the surface of worms, while nontreated adult worms showed intact surface (Figure 1).

*Schistosoma*' tegument of is well-recognized as an important drug target and model of study in schistosomiasis [41]. In this regard, tegument is pivotal for the survival of *Schistosoma* not only because it is one of the major route for nutrient absorption, but also because it is important for the protection of schistosomes, since tegumental changes might result in exposure of parasite antigens to the host immune system [41, 42].

**Figure 2:**
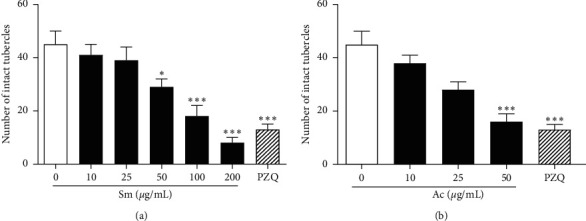
Effect of Sm and Ac on tubercles of male schistosomes. Quantification was performed using confocal microscopy. Intact tubercles was measured in a 20,000 *μ*m^2^ of area calculated with the Zeiss LSM Image Browser software. Praziquantel (PZQ, 2 *μ*M) was used as reference compound. A minimum of three tegument areas of each parasite were assessed. Values are mean ± SD (bars) of ten male adult worms. ^*∗*^*P* < 0.05 and ^*∗∗∗*^*P* < 0.001 compared with untreated groups.

Furthermore, a quantitative analysis of the number of intact tubercles on male parasites was performed (Figure 2). Results showed dose-response effects by Sm (Figure 2(a)) and Ac (Figure 2(b)) on the tubercles of the male worm teguments. Remarkable, after exposure to 200 *μ*g/mL of Sm (Figure 2(a)) a reduction was observed in the intact tubercles of 82.2% (*P* < 0.001), while PZQ (5 *μ*M) and Ac (50 *μ*g/mL) caused 71.1% (*P* < 0.001) and 64.4% (*P* < 0.001) of reduction.

**Figure 3:**
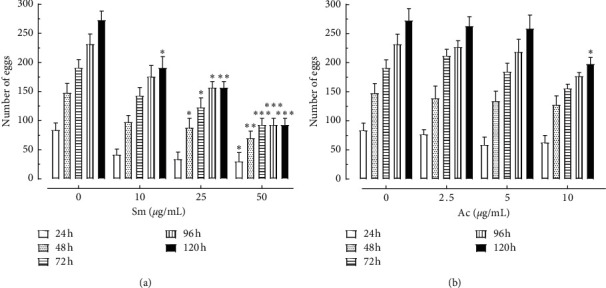
Effect of Sm and Ac on oviposition of *S*. *mansoni*. Adult worm couples were incubated with nonlethal concentrations of Sm and Ac and, at the indicated time periods, and the cumulative number of eggs was assessed using an inverted microscope. Values are mean ± SD (bars) of ten worm couples. ^*∗*^*P* < 0.05, ^*∗∗*^*P* < 0.01, and ^*∗∗∗*^*P* < 0.001 compared with untreated groups.

In addition, the schistosomicidal activity can also be assessed by the ability of samples in suppressing female oviposition [43]. Regarding egg production, groups of parasites were incubated with Sm and Ac, and the number of eggs by adult worms of *S*. *mansoni* was monitored for 120 hours (Figure 3). The egg production in *S*. *mansoni* adult females was inhibited significantly after 48 hours exposure of Sm (25–50 *μ*g/mL) and after 120 hours with Ac (10 *μ*g/mL) (Figure 3). Following 120 hours exposure with Sm (50 *μ*g/mL) and Ac (10 *μ*g/mL), egg laying was decreased significantly in 65.9% and 27.5% in comparison to the negative control group. Results showed a suppression of egg laying in all sublethal concentrations of Sm and Ac. These are important observed antischistosomal effects, since the pathology of human schistosomiasis is directly associated to the large number of eggs, which become trapped in the hosts tissues, resulting in immunopathological lesions that are characterized by inflammation and fibrosis in the target organs [44]. Other plant extracts active on the sexual reproductive fitness of schistosomes are from the leaves of *Clerodendrum umbellatum* (Verbenaceae) [45] and from the roots of *Zingiber officinale* (Zingiberaceae) [46].

**Figure 4:**
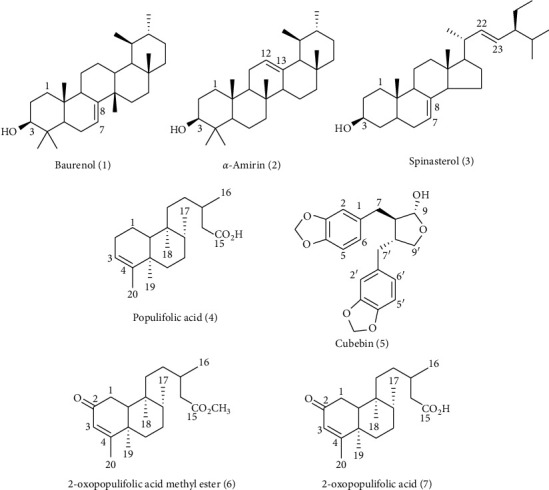
Chemical structures of compounds isolated from the crude extracts of *S*. *microglossa* (**1**–**3**) and *A*. *cymbifera* (**4**–**7**).

Additionally, Sm was submitted to chromatographic fractionation, yielding three isolated compounds (Figure 4), which were chemically identified by ^13^C- and ^1^H- NMR data analysis in comparison to those in the literature as follows: baurenol (**1**) [24], *α*-amirin (**2**) [25], and spinasterol (**3**) [26]. Similarly, populifolic acid (**4**) [27], cubebin (**5**) [28], 2-oxopopulifolic acid methyl ester (**6**) [29–31], and 2-oxopopulifolic acid (**7**) [29–31] were isolated after chromatographic fractionation of Ac (Figure 4). Purity of all isolated compounds was predictable to be higher than 95% by ^13^C- and ^1^H- NMR data analysis.

All isolated compounds (25–100 *μ*M) were also evaluated against *S*. *mansoni* adult worms (Table 2). Regarding compounds from Sm, bauerenol (**1**), *α*-amirin (**2**), and spinasterol (**3**) were able to decrease the motor activity of adult schistosomes in a dose-dependent manner (Table 1), without causing lethal effects on schistosomes, even when tested at 100 *μ*M. Although bauerenol (**1**), *α*-amirin (**2**), and spinasterol (**3**) were not lethal to schistosomes, they showed significant impact on worm motor activity of *S*. *mansoni*, like the crude Sm extract.

Furthermore, along with the isolated compounds from Ac, populifolic acid (**4**) and cubebin (**5**) were not lethal at concentrations of 25–100 *μ*M but caused significant reduction in motor activity and movements of parasites (Table 2). Populifolic acid (**4**) and cubebin (**5**) have been previously isolated from *A*. *cymbifera* [18, 29], while populifolic acid (**4**) is a diterpene widely found in *Aristolochia* species [18]. In addition, cubebin (**5**) is a lignan previously evaluated against *S*. *mansoni*. Recently, Parreira et al., showed that cubebin (**5**) (at 100 *μ*M) can separate adult worm pairs and reduce egg laying, without causing death in adult schistosomes [47]. PZQ (2 *μ*M) was lethal to 100% of the worms after 24 h of incubation, while all worms incubated in RPMI-1640 stayed alive until the end of the experiment.

On the other hand, 2-oxopopulifolic acid methyl ester (**6**) and 2-oxopopulifolic acid (**7**) showed the best in vitro antischistosomal activity, causing death and decrease of motor activity in all adult schistosomes at 100 *μ*M after 24 h of incubation (Table 2). Results suggested dose-response effects by 2-oxopopulifolic acid methyl ester (**6**) and 2-oxopopulifolic acid (**7**) on the mortality rate of schistosomes (Table 2). 2-Oxopopulifolic acid methyl ester (**6**) and 2-oxopopulifolic acid (**7**) have been previously isolated from *Aristolochia* species [18]. Previous report showed that 2-oxopopulifolic acid (**7**) isolated from *A*. *cymbifera* showed antimicrobial activity against *Staphylococcus sp* and *Pseudomonas aeruginosa* [48, 49].

Diterpenes are a class of plant-derived compounds that display a broad spectrum of biological activities, including antiparasitic effects against parasites of neglected tropical diseases [50]. Previous reports show that some diterpenes possess schistosomicidal activity against *S*. *mansoni* [33], such as pimaradienoic acid [51], isolated from *Viguiera Arenaria* (Asteraceae) and 7-ceto-sempervirol, obtained from *Lycium chinense* [52]. Also, although the knowledge about the schistosomicidal properties of diterpenes is limited, the scientific literature has pointed out that some diterpenes may be potentially employed as prototypes for further in vitro and in vivo investigations against *S*. *mansoni*, such as the diterpene phytol [32] and other acid diterpenes from *Copaiba* species [53].

The schistosomicidal results of methyl-2-oxopopulifoloate (**6**) and 2-oxopopulifolic acid (**7**) suggest that these diterpenes may be important candidates for further antischistosomal investigations.

## 4. Conclusion

In this study, we have reported, for the first time, the in vitro antischistosomal effects of *S*. *microglossa* and *A*. *cymbifera* extracts, with no cytotoxicity on mammalian cells. Also, we have isolated and identified compounds from these active extracts that demonstrate in vitro properties against adult schistosomes. Finally, our findings identified some diterpenes as promising lead antischistosomal compounds to further antiparasitic investigations.

## Figures and Tables

**Table 1:** In vitro schistosomicidal and cytotoxic activities of Sm and Ac.

Groups	Dead worms (%)^a,c^	Motor activity reduction (%)^a^		Worms with tegumental alterations (%)^a^		Cytotoxicity CC_50_ (*μ*g/mL)^d^
		Slight	Significant	Partial	Extensive	
Control^*b*^	—	—	—	—	—	—
PZQ (2 *μ*M)	100	—	100	—	100	—
DMSO 0.5%	—	—	—	—	—	—
Sm^c^						>200
200	100	—	100	—	100	
100	50	—	100	—	100	
50	—	50	50	50	50	
25	—	50	50	50	50	
10	—	—	—	—	—	
Ac^c^						>200
200	100	—	100	—	100	
100	100	—	100	—	100	
50	50	50	50	50	50	
25	25	50	50	50	50	
10	—	25	—			

^a^Percentages relative to the 20 worms investigated. ^b^RPMI 1640. ^c^Incubation period: 24 h with concentrations in *μ*g/m. ^d^CC_50_ values (50% cytotoxicity concentration) on Vero cells.

**Table 2:** *In vitro* schistosomicidal and cytotoxic activities of isolated compounds from Sm and Ac.

Groups	Dead worms (%)^a,^	Motor activity reduction (%)^a^		Worms with tegumental alterations (%)^a^		Cytotoxicity CC_50_ (*μ*M)^d^
		Slight	Significant	Partial	Extensive	
Control^*b*^	—	—	—	—	—	—
PZQ^*c*^ 2 *μ*M	100	—	100	25	75	—
DMSO 0.5%	—	—	—	—	—	—
		Compounds^c^				
*α*-Amirin (**1**)						>100
100	—	—	100	—	—	
50	—	50	50	—	—	
25	—	—	—	—	—	
Baurenol (**2**)						>100
100	—	—	100	—	—	
50	—	50	50	—	—	
25	—	—	—	—	—	
Spinasterol (**3**)						>100
100	—	100	—	—	—	
50	—	—	—	—	—	
25	—	—	—	—	—	
Populifolic acid (**4**)						>100
100	—	—	100	—	—	
50	—	—	—	—	—	
25	—	—	—	—	—	
Cubebin (**5**)						>100
100	—	—	100	—	—	
50	—	50	50	—	—	
25	—	50	50	—	—	
2-oxopopulifolic acid methyl ester (**6**)						>100
100	100	—	100	100	—	
50	50	—	100	—	—	
25	—	50	—	—	—	
2-oxopopulifolic acid (**7**)						>100
100	100	—	100	100	—	
50	50	—	100	—	—	
25	—	50	—	—	—	

^a^Percentages relative to the 20 worms investigated. ^b^RPMI 1640. ^c^Incubation period: 24 h, tested at *μ*M. ^d^CC_50_ values CC_50_ values (50% cytotoxic concentration) on Vero cells.

## Data Availability

The data used to support the findings of this study are included within the article.
